# Association of intraosseous and intravenous access with patient outcome in out-of-hospital cardiac arrest

**DOI:** 10.1038/s41598-023-48350-8

**Published:** 2023-11-27

**Authors:** Frederik Nancke Nilsson, Søren Bie-Bogh, Louise Milling, Peter Martin Hansen, Helena Pedersen, Erika F. Christensen, Jens Stubager Knudsen, Helle Collatz Christensen, Fredrik Folke, David Høen-Beck, Ulla Væggemose, Anne Craveiro Brøchner, Søren Mikkelsen

**Affiliations:** 1 The Prehospital Research Unit, Department of Regional Health Research, University of Southern Denmark, Odense, Denmark; 2 OPEN, Open Patient Data Explorative Network, University of Southern Denmark, Odense, Denmark; 3 Department of Cardiology, North Zealand Hospital, Hillerød, Denmark; 4 The Danish Air Ambulance, Aarhus, Denmark; 5 Department of Clinical Medicine, Centre for Prehospital and Emergency Research, Aalborg University Hospital, Aalborg University, Aalborg, Denmark; 6 Department of Clinical Medicine, Aalborg University, Aalborg, Denmark; 7 Department of Anaesthesiology, Kolding University Hospital, Kolding, Denmark; 8 Prehospital Center, University of Copenhagen, Naestved, Denmark; 9 Department of Clinical Medicine, University of Copenhagen, Copenhagen, Denmark; 10 Danish Clinical Quality Program (RKKP), National Clinical Registries, Copenhagen, Denmark; 11 Copenhagen Emergency Medical Services, University of Copenhagen, Ballerup, Denmark; 12 Department of Cardiology, Herlev Gentofte University Hospital, Copenhagen, Denmark; 13 Department of Anaesthesiology, Denmark and Prehospital Center, Holbæk Hospital, HolbækRegion Zealand, Denmark; 14 Department of Research and Development, Prehospital Emergency Medical Services, Central Denmark Region, Aarhus, Denmark; 15 Department of Clinical Medicine, Aarhus University, Aarhus, Denmark; 16 Department of Anesthesiology and Intensive Care, Odense University Hospital, Odense, Denmark; 17 The Prehospital Research Unit, Region of Southern Denmark, Odense University Hospital, 5000 Odense C, Denmark

**Keywords:** Diseases, Cardiovascular diseases

## Abstract

Here we report the results of a study on the association between drug delivery via intravenous route or intraosseous route in out-of-hospital cardiac arrest. Intraosseous drug delivery is considered an alternative option in resuscitation if intravenous access is difficult or impossible. Intraosseous uptake of drugs may, however, be compromised. We have performed a retrospective cohort study of all Danish patients with out-of-hospital cardiac arrest in the years 2016–2020 to investigate whether mortality is associated with the route of drug delivery. Outcome was 30-day mortality, death at the scene, no prehospital return of spontaneous circulation, and 7- and 90-days mortality. 17,250 patients had out-of-hospital cardiac arrest. 6243 patients received no treatment and were excluded. 1908 patients had sustained return of spontaneous circulation before access to the vascular bed was obtained. 2061 patients were unidentified, and 286 cases were erroneously registered. Thus, this report consist of results from 6752 patients. Drug delivery by intraosseous route is associated with increased OR of: No spontaneous circulation at any time (OR 1.51), Death at 7 days (OR 1.94), 30 days (2.02), and 90 days (OR 2.29). Intraosseous drug delivery in out-of-hospital cardiac arrest is associated with overall poorer outcomes than intravenous drug delivery.

## Introduction

Out-of-hospital cardiac arrest (OHCA) is a general healthcare issue with a high mortality rate and a large burden for healthcare organisations^1^. In Denmark, up to 5000 people suffer from OHCA each year^2^ and 14% survive for 30 days. Treatment of OHCA consists of a time-critical sequence of interventions^3^. Advanced life support includes adrenaline for patients with pulseless electric activity (PEA) or asystole and amiodarone combined with adrenaline for patients with shock-refractory ventricular fibrillation or pulseless ventricular tachycardia^4–8^. When choosing the route of administration for these drugs, the prehospital clinician has several options^4^ including intraosseous or intravenous access.

Current resuscitation guidelines recommend the use of intravenous access as the first choice when vascular access is required during cardiopulmonary resuscitation (CPR)^4,9,10^. The same guidelines encourage the resuscitation provider to consider intraosseous access if attempts at intravenous access are unsuccessful or intravenous access is not feasible^4^. Establishing intravenous access can be challenging in OHCA because of the compromised circulation, the difficult work conditions in the out-of-hospital setting, and the urgency of the procedure. Intraosseous access provides fast access to the circulatory system and has a high success rate^11–14^. Previous studies have indicated poorer outcomes for patients treated with intraosseous access when compared to an intravenous access^15–20^. In a randomized study, Daya et al.^19^ investigated patients who were randomised to lidocaine, amiodarone, or placebo and found that the point estimates for the effects of both drugs in comparison with placebo were significantly greater for the intravenous than for the intraosseous. Until now, no consensus has been established on whether intraosseous access equates to intravenous access.

In this retrospective study we compare the outcomes of patients with OHCA treated via an intraosseous access with patients with OHCA treated through an intravenous access. The outcomes are: 30-day mortality, no prehospital return of spontaneous circulation, patient pronounced dead at the scene, 7-day mortality, and 90-day mortality.

## Methods

### System setting

Denmark has a population of approximately 5.8 million people. The emergency medical system is a tax-funded three-tiered system providing services without direct costs to the patient^21^. For simple emergencies, an ambulance manned with emergency medical technicians (EMT) and/or paramedics is dispatched. In more complicated cases, for example, OHCA, both an ambulance and a rapid response vehicle with a paramedic or a Mobile Emergency Care Unit manned by a specialist in anaesthesiology^22^ are dispatched to the site. In cases where the site of emergency is in a remote area, the Helicopter Emergency Medical Service may be dispatched^23^. During and immediately after the treatment of a patient, the attending EMT, paramedic, and the prehospital anaesthesiologist all register the findings and the treatment administered in the nationwide prehospital medical records^22^.

### Design and data sources

In this retrospective cohort study, we used data from two national healthcare registers and national prehospital medical records^22^. Data regarding all Danish OHCA patients from January 1, 2016, to December 31, 2020, were collected from The Danish Cardiac Arrest Registry^2,24^. Subsequently, general health data were collected using the unique patient identifier the Danish Civil Personal register number assigned to all Danish residents^25^.

The following variables were collected from The Danish Cardiac Arrest Registry:

Age, sex, date of the OHCA, information whether the OHCA was witnessed by bystanders or not, the provision of basic life support initiated before the arrival of an ambulance, the provision of defibrillation by a bystander, the provision of defibrillation by ambulance personnel, the first observed cardiac rhythm (dichotomised as shockable or non-shockable), the patient´s status at arrival to hospital (stratified into four groups: resuscitation terminated, ongoing resuscitation, unconscious patient with spontaneous circulation or conscious patient with spontaneous circulation) and response time of the first prehospital vehicle.

We collected information on pharmacological access route for drugs (intraosseous or intravenous) from the national prehospital medical record system^22^. This information was gathered from formalised registration boxes in which the prehospital provider ticks the route of administration of drugs (i.e., intravenous administration, intraosseous administration). Furthermore, all text fields were scrutinized for written indications of the use of an intraosseous access (i.e., words containing "intraosseous", "osseous", the abbreviation "IO)".

The Danish Civil Registration System provided information on dates of death^25^. No distinctions were made regarding the causes of death.

### Outcomes

Five parameters were included in the analysis. The primary outcome was “30-day mortality (I)”. The secondary outcomes were “No ROSC at any point during treatment” (II), “Death at the scene (III)”, “7-day mortality (IV)”, and “90-day mortality (V)”.

### Statistical analysis

The predictor variables were compared in the two groups using a Pearson’s chi-squared test for categorical variables and either a Wilcoxon rank-sum or student t-test for continuous variables. The associations between intraosseous access or intravenous access and the outcomes were calculated using logistic regression. Both crude and adjusted analyses were conducted. All analyses used clustered robust standard errors to consider that the same person may be included multiple times. The adjusted analysis included factors known to impact the outcome variables. The analyses were adjusted for age, sex, witnessed OHCA or not, basic life support provided before ambulance arrival, bystander defibrillation, defibrillation administered by ambulance personnel, ambulance response time, and shockable/non-shockable rhythm^26^. A 5% significance level was applied. Results are presented as Odds-Ratios (OR). Data management and analyses were performed using Stata 16 (Stata Corp LP, College Station, Texas, USA).

### Ethical approvals

All research was performed in accordance with relevant guidelines and regulations. The project was approved by the Danish Patient Safety Authorities (Ref. No. 31-1521-434). According to the Act on Processing of Personal Data, in register-based studies approved by the Danish Patient Safety Authorities, no consent for the use of data already deposited in the registry is required. No further approvals are necessary according to Danish law and accordingly, the requirement for informed consent was waived^27^. In addition to the necessary approvals being obtained, all data handling was carried out respecting the Danish and European legislation concerning person-identifiable data^28,29^.

## Results

**Figure 1:**
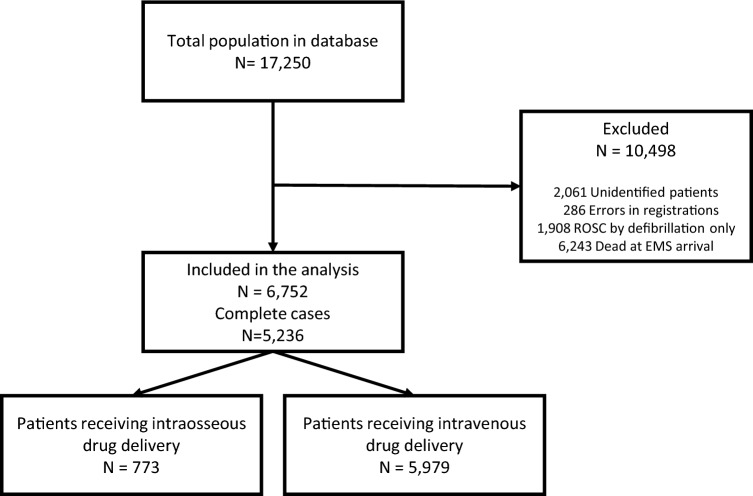
Flowchart.

In total, 17,250 cardiac arrest patients were included in the study. As illustrated in Fig. 1, 6243 patients were declared dead at the scene without treatment by the attending prehospital anaesthesiologist. 1908 patients had return of spontaneous circulation (for example as a result of the defibrillation) before an access to the vascular bed was procured. In 2061 cases, no reliable personal identification was obtained, and 286 cases were excluded because of incomplete data. Overall, 6752 patients were included in the statistical analysis. Of these, 773 (11.4%) received an intraosseous access, and 5979 (88.6%) received an intravenous access. The latter group of patients were on average 3 years older (70 years) than patients in the intraosseous access group (67 years) (*p* < 0.001, Student´s t-test). The intravenous access group contained significantly more patients with a shockable rhythm as the first observed rhythm compared with the intraosseous access group (20.7% vs. 14.5%, *p* < 0.001, Pearson´s Chi^2^ test).

Our primary outcome, 30-day mortality, was 90.5% for all patients included in the study. 30-day mortality were higher in the intraosseous access group than in the intravenous access group (95.3% vs. 89.9%, *p* < 0.001, Pearson´s Chi^2^ test). Patients in the intraosseous access group were less prone to achieve ROSC than patients in the intravenous access group (No-ROSC: 72.1% vs. 62.4%, *p* < 0.001, Pearson´s Chi^2^ test). Significantly more patients in the intraosseous group were declared dead at the scene following EMS treatment than in the intravenous access group (83.4% vs. 80.6%, *p* < 0.001, Pearson´s Chi^2^ test). The 7-day mortality was higher in the intraosseous access group than in the intravenous access group (93.8% vs. 87.1%, *p* < 0.001, Pearson´s Chi^2^ test). 90-day mortality was higher in the intraosseous group than the intravenous access group (96.1% vs. 90.4%, *p* < 0.001, Pearson´s Chi^2^ test). See Table 1 for details.

**Table 1:** Danish out-of-hospital-cardiac arrest patients 2016—2020. Demographics and outcomes.

Variable	Total	Intravenous	Intraosseous	*p*-value
	N = 6752	N = 5979	N = 773	
Male sex, n (%)	4459 (66.0%)	3956 (66.2%)	503 (65.1%)	0.56^a^
Mean age, years (SD)	69 (15.49)	70 (15.30)	67 (16.71)	< 0.001^b^
Witnessed events, n (%)	3219(47.7%)	2869 (48.0%)	350 (45.4%)	0.17^a^
Basic life support before ambulance arrival, n (%)	4712 (69.8%)	4178 (69.9%)	534 (69.2%)	0.67^a^
Defibrillation given by bystander, n (%)	544 (8.1%)	498 (8.3%)	46 (6.0%)	0.023^a^
Defibrillation given by ambulance personnel, n (%)	2283 (33.8%)	2079 (34.8%)	204 (26.5%)	< 0.001^a^
Response time, median (IQR)	7 (5–10)	7 (5–10)	7 (5–10)	0.096^c^
First observed rhythm is shockable, n (%)	1348 (20.0%)	1236 (20.7%)	112 (14.5%)	< 0.001^a^
NO Return of spontaneous circulation, n (%)	4288 (63.5%)	3731 (62.4%)	557 (72.1%)	< 0.001^a^
Dead at the scene following EMS administrated treatment, n (%)	5478 (81.1%)	4818 (80.6%)	660 (83.4%)	< 0.001^a^
7-day mortality, n (%)	5933 (87.9%)	5208 (87.1%)	725 (93.8%)	< 0.001^a^
30-day mortality, n (%)	6112 (90.5%)	5375 (89.9%)	737 (95.3%)	< 0.001^a^
90-day mortality, n (%)	6149 (91.1%)	5406 (90.4%)	743 (96.1%)	< 0.001^a^
Resuscitation terminated at arrival to hospital*, n (%)	3946 (58.4%)	3425 (57.3%)	521 (67.4%)	< 0.001^a^
Ongoing CPR at arrival to hospital*, n (%)	868 (12.9%)	782 (13.1%)	86 (11.1%)	
Tactile pulse/other signs of life at arrival to hospital*, n (%)	1525 (22.6%)	1386 (23.2%)	139 (18.0%)	
GCS > 8 at arrival to hospital*, n (%)	375 (5.6%)	355 (5.9%)	20 (2.6%)	
Missing	31 (0.5%)	24 (0.4%)	7 (0.9%)	

Data are presented as mean (SD) or median (IQR) for continuous measures, and n (%) for categorical measures.

*GCS* glasgow coma score, *CPR* cardiopulmonary resuscitation.

*Not all patients were transported to hospital following termination of resuscitation.

^a^Pearson´s Chi^2^ test.

^b^Student´s t-test.

^c^Wilcoxon rank-sum test.

### Adjusted analyses

The outcome variables were analysed with independent variables that are known predictors for survival of OHCA^26^. Apart from the variable “Dead at the scene” which did not differ when adjusted for confounders, for all variables, the use of an intraosseous access to the vascular bed was associated with poorer outcomes than the use of an intravenous access with a crude OR of being dead at 30 days of 2.28 (95% CI 1.62; 3.22) and an adjusted OR of 2.02 (95% CI 1.34; 3.05). For crude and adjusted OR for the outcome variables “Dead at the scene”, “No return of spontaneous circulation”, “Dead at the scene”, “7-day mortality”, and “90-day mortality”, see Table 2.

**Table 2:** Association of No ROSC, Dead at the scene, 7-day mortality, 30-day mortality, and 90-day mortality, intraosseous administration of drugs; intravenous administration of drugs as reference.

Intravenous access	Reference	Crude OR		Adjusted OR	
		1		1	
			*P*-value^a^	OR	*P*-value^a^
Intraosseous access	No ROSC	1.55 (1.31; 1.83)	< 0.001	1.51 (1.23; 1.84)	< 0.001
	Dead at the scene	1.47 (1.19; 1.82)	< 0.001	1.28 (0.96; 1.61)	0.102
	7-day mortality	2.22 (1.64; 3.00)	< 0.001	1.94 (1.34; 2.79)	0.001
	30-day mortality	2.28 (1.62; 3.22)	< 0.001	2.02 (1.34; 3.05)	0.001
	90-day mortality	2.60 (1.79; 3.79)	< 0.001	2.29 (1.47; 3.56)	0.001

Crude Odds-Ratio and Odds-Ratio adjusted for sex, age, witnessed cardia arrest, basic life support before ambulance arrival, defibrillation given by bystander, defibrillation given by ambulance personnel, response time.

^a^ All *p*-values derived from logistic regression analyses.

## Discussion

In this retrospective, observational study comparing intraosseous versus intravenous access in OHCA the main findings were: (1) a significant increase in mortality following OHCA in patients treated with drug delivery through an intraosseous access than in patients treated through an intravenous access, (2) a more than two-fold increased odds ratio of dying within 30 days following an OHCA when treated via an intraosseous access compared with treatment via an intravenous access.

The current guideline on resuscitation suggests that the same drug doses are used regardless of the administration site being an intravenous access or an intraosseous access^4,10^. The effect of amiodarone and adrenaline used in treatment of OHCA has been described in previous studies^30–32^. Although adrenaline is considered to increase the likelihood of achieving ROSC and may lead to improved overall survival, it is still uncertain if the increased number of patients with ROSC comes at a cost of an increase in the number of patients with neurological injury^5,31,33–35^. As most of the studies on the subject have not investigated the administration site, it is less certain what the effects are when the drugs are administered intraosseously. Despite the low evidence on the subject, intraosseous access is frequently used in the acute setting, as it provides fast, non-collapsible access to the venous plexus in the bone marrow, with a higher first-attempt success rate than intravenous access^14,36^. A randomised controlled trial (RCT) investigated administration of amiodarone, adrenaline, and placebo, via intravenous or intraosseous access in OHCA using neurological outcome and survival as outcomes. Overall, neither amiodarone nor lidocaine resulted in a significantly higher rate of survival or favourable neurologic outcome^15^. The study did not address the site of administration. However, a sub-analysis of the results revealed that point estimates for the effects of both drugs compared to placebo were greater for the intravenous than intraosseous route across all outcomes and beneficial only for intravenous^19^. Further, a recent retrospective study from Germany including 212, 228 OHCA patients over 31 years also found an association between poorer clinical effect of intraosseous adrenaline in OHCA^20^. All these studies support our findings that intraosseous access compared with intravenous access might be associated with a higher OR of mortality, not all studies have shown the drug administration route to be relevant in OHCA. One retrospective study investigated the first access site attempted. Based on the outcome ROSC at hospital arrival, the authors reported that a first intraosseous access approach was non-inferior to a first intravenous approach^37^. A common trait in these studies is the potential for confounding by indication. Intuitively, the intravenous access to the vascular bed is the primary target for gaining access to the vascular bed. There are undoubtedly cases in which the prehospital clinician only resort to an intraosseous access once intravenous access has proven impossible. Indeed, the figures found in our study (773 patients with an intraosseous access and 5979 patients with an intravenous access), it should be obvious that the first attempt to gain access to the vascular bed most often is an intravenous access. This may result in “resuscitation time bias” as the intraosseous access group may, inherently, have had a delay in drug administration resulting in time spent trying to insert an intravenous access before resorting to intraosseous access^38^.

As these studies are based on retrospective analyses, there is no possibility to adjust for this potential confounding effect. There is only limited knowledge on the effect of administration site and the outcomes “survival” or “favourable neurological outcome”. Reports from several clinical studies vary with findings of both similar outcomes regardless of access route^39–41^ and differing outcomes^16–19,42,43^ when comparing intraosseous drug delivery and intravenous drug delivery in OHCA patients.

Animal studies comparing intravenous and intraosseous administration of amiodarone in cardiac arrest have produced conflicting results regarding both clinical outcomes and pharmacokinetic outcomes, for example time to peak concentrations (${T}_{max}$)^44^. One animal study suggested that a higher dose of intraosseous adrenaline might be needed to generate coronary perfusion pressure values similar to what could be achieved using standard doses intravenously during resuscitation in prolonged ventricular fibrillation^45^. Other studies comparing intraosseous and intravenous administration of drugs in porcine models have reported a difference in peak serum concentration (${C}_{\mathrm{max}}$) values; intraosseous adrenaline not reaching concentrations equalling the concentrations obtained in intravenous treatment^46,47^. If the described findings concerning mechanisms of action are transferable from animals to humans, this could explain our findings of an association of a poorer outcome following resuscitation with drugs administered intraosseously, as the concentration of the drugs given through an intraosseous access might be too low to achieve the same effect as when given through an intravenous access.

The main limitation of our study is the risk of confounding by indication. To some clinicians intraosseous access may be considered an emergency procedure to revert to only after intravenous access fails. Insertion of an intraosseous cannula may be caused by the clinician´s inability to insert an intravenous access. One or more failed attempts to place an intravenous access before reverting to an intraosseous access may thus cause a delay in the medical treatment. The mere presence of an intraosseous access may even reflect the possibility that access to the vascular bed may have taken priority and maybe even delayed basic life support, such as chest compression and/or rescue breaths. This phenomenon, called resuscitation time bias may limit our conclusions^38^.

On the other hand, there is a notion among Danish clinicians that in cases where the rapid response vehicle with only one paramedic is first at the scene, the intraosseous access is widely used as it is easier and faster than attempts at establishing an intravenous access^48^. As our study is a register-based study, we have no possibility to adjust for these potential confounders and we have no data allowing us to discriminate between patients in whom the intraosseous access was the first choice and patients that had an intraosseous access placed after a failed intravenous attempt.

Although other retrospective studies have the same limitations in interpreting the results, our findings of a poorer outcome being associated with intraosseous access are supported by previous studies, which indicate a poorer outcome for patients treated through an intraosseous access^16–19,40,42,43^.

One limitation of this study is that the group of patients having intravenous access placed during their treatment was significantly younger and to a larger extent had shockable rhythm. However, we adjusted for these two variables in our analysis of Odds-Ratios. Another apparent limitation of this study is the large number of patients excluded from analysis despite initial inclusion into the Danish National Cardiac Arrest Registry^2,24^. 6243 patients were in effect dead but had been subjected to resuscitation attempts by lay persons. As the criterion defining a patient´s eligibility for being registered in the registry is “any basic life support administered prehospitally”, these patients were registered in the Danish National Cardiac Arrest Registry. They were, however, declared dead at the arrival of the prehospital physician and no treatment was thus initiated. 1908 patients had return of spontaneous circulation following defibrillation before any vascular access had been attempted. These patients were excluded from the analysis. In 2061 cases, the unique civil personal register number of the patient was not known at the time of registering the patient. These patients were lost to follow-up. In 286 cases, errors in registration, for example, a patient without a cardiac arrest being erroneously registered as dead, were apparent. These two latter groups, comprising 2367 patients, constitute the real limitation of the study.

Another limitation in the present study was that we could not differentiate between tibial intraosseous access or humeral intraosseous access or assess any use of a pressurised fluid bag when using intraosseous access. This could have influenced the efficiency of the drugs^46,47,49,50^.

Although our results should be interpreted with caution, our findings suggest that the survival of OHCA patients is associated with the method that the prehospital personnel uses to gain access to the vascular bed. Administration of drugs through intraosseous access is associated with poorer outcome in almost all the parameters that are usually reported in OHCA. It is possible that the clinical practice of applying intraosseous access should be reviewed.

## Data Availability

Anonymised data are available from the corresponding author on reasonable request.
